# Supplementary figures and images for: Genome sequencing and CAZymes repertoire analysis of Diaporthe eres P3-1W causing postharvest fruit rot of ‘Hongyang’ kiwifruit in China
Source: PeerJ. 2024 Aug 5;12:e17715. doi: 10.7717/peerj.17715 (PMC11308996; doi:10.7717/peerj.17715)

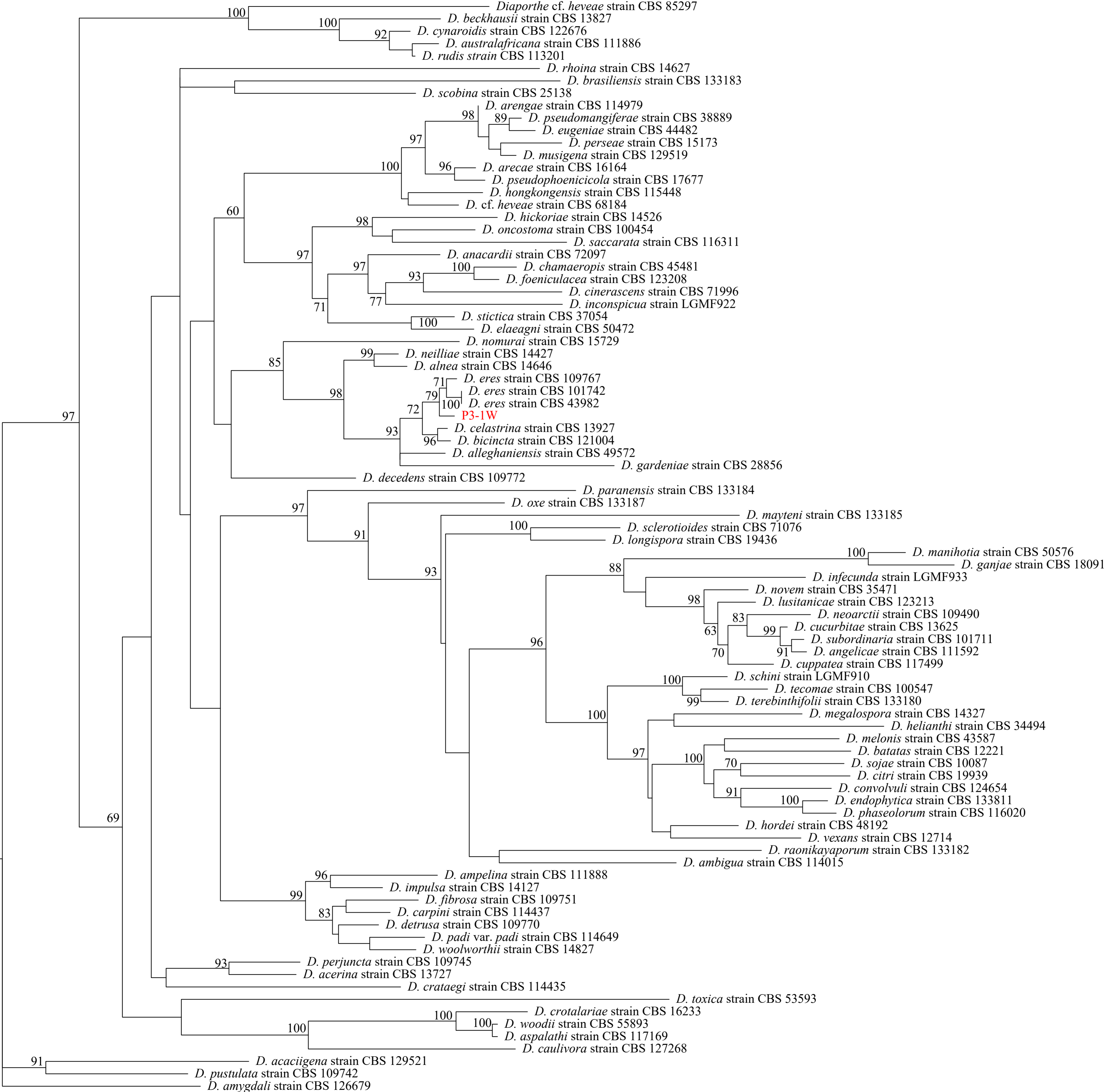

0.05

Supplement: Supplemental Information 1 [file peerj-12-17715-s001.pdf]

# Gene Function Classification (GO)

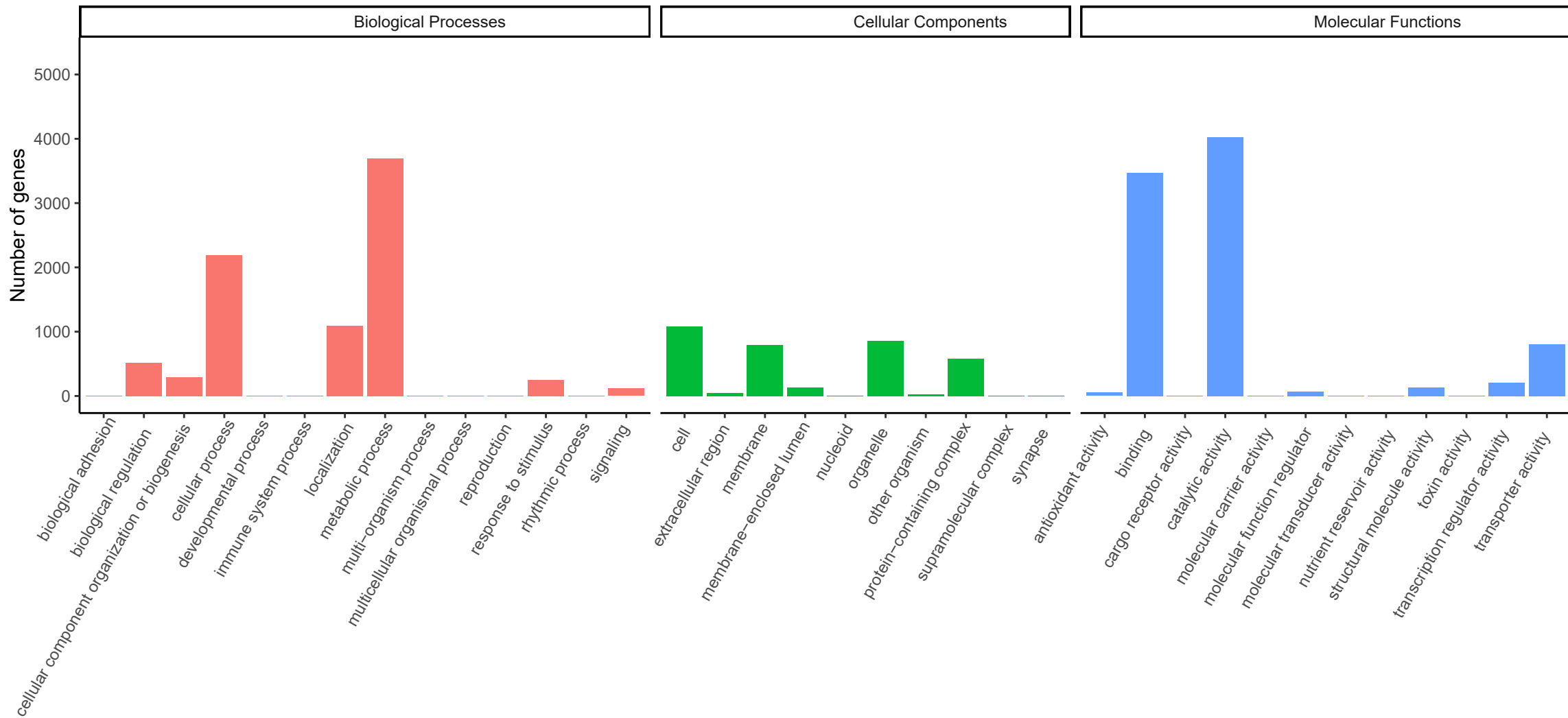

Supplement: Supplemental Information 2 [file peerj-12-17715-s002.pdf]

KEGG Classification

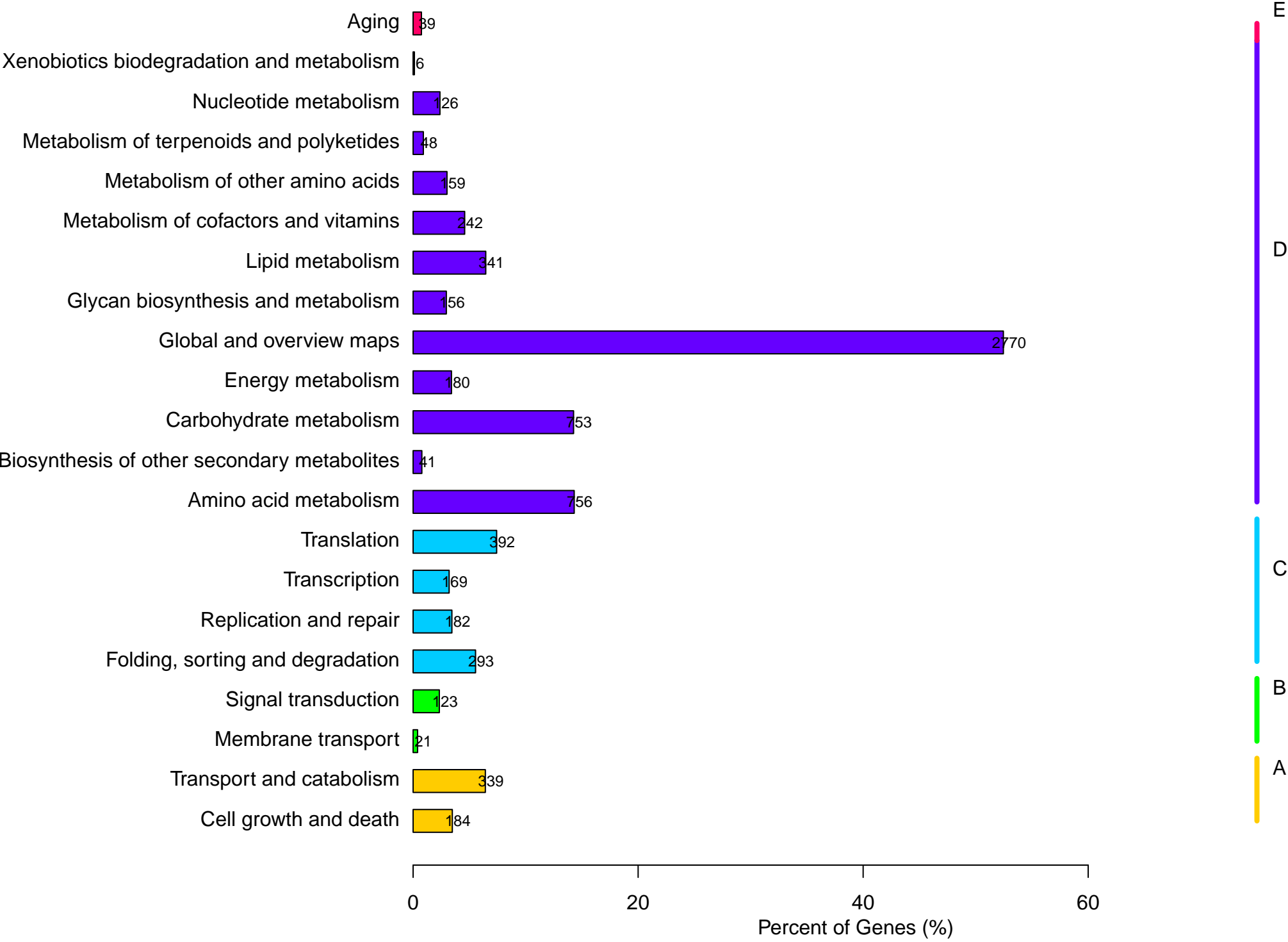

Supplement: Supplemental Information 3 [file peerj-12-17715-s003.pdf]
