# Supplementary material for: Genome sequencing and CAZymes repertoire analysis of Diaporthe eres P3-1W causing postharvest fruit rot of ‘Hongyang’ kiwifruit in China
Source: PeerJ. 2024 Aug 5;12:e17715. doi: 10.7717/peerj.17715 (PMC11308996; doi:10.7717/peerj.17715)
Supplement: Supplemental Information 4 [file peerj-12-17715-s004.docx]

**Table S1.** Primer sets and corresponding amplification targets

| Target gene | Primer | Primer DNA Sequence (5‘-3’) |
| --- | --- | --- |
| ITS | ITS1 | TCCGTAGGTGAACCTGCGG |
|  | ITS4 | TCCTCCGCTTATTGATATGC |
| TEF1α | EF1-728F | CATCGAGAAGTTCGAGAAGG |
|  | EF1-986R | TACTTGAAGGAACCCTTACC |
